# Within-population genetic diversity and population structure of *Plasmodium knowlesi* merozoite surface protein 1 gene from geographically distinct regions of Malaysia and Thailand

**DOI:** 10.1186/s12936-018-2583-z

**Published:** 2018-11-29

**Authors:** Md Atique Ahmed, Ki-Back Chu, Indra Vythilingam, Fu-Shi Quan

**Affiliations:** 1 Department of Medical Zoology, School of Medicine, Kyung Hee University, Seoul, 02447 Republic of Korea; 2 Department of Biomedical Science, Graduate School, Kyung Hee University, Seoul, 02447 Republic of Korea; 3 Parasitology Department, University of Malaya, Kuala Lumpur, Malaysia; 4 Biomedical Science Institute, Kyung Hee University, Seoul, 02447 Republic of Korea

**Keywords:** Merozoite surface protein 1, Natural selection, Vaccine, Genetic diversity, Sub-populations, *Plasmodium knowlesi*

## Abstract

**Background:**

The C-terminal 42 kDa domain of *Plasmodium knowlesi* merozoite surface protein 1 (PkMSP1) is a potential asexual blood-stage vaccine candidate, however, only a limited number of clinical isolates have been analysed from Malaysia and no inter-country comparative diversity study has been conducted. In the present study, nucleotide diversity, haplotypes and natural selection levels of *pkmsp1* in clinical samples from geographically distinct regions of Malaysia and Thailand were investigated. The overall population structure of the parasite from the region was determined.

**Methods:**

Eleven full-length *pkmsp1* sequences obtained from clinical isolates of Malaysia along with the H-strain were downloaded from the database for domain wise characterization of *pkmsp1* gene. Additionally, 76 *pkmsp*-*1*_*42*_ sequences from Thailand and Malaysia were downloaded from the database for intra and inter-population analysis. DnaSP 5.10 and MEGA 5.0 software were used to determine genetic diversity, polymorphism, haplotypes and natural selection. Genealogical relationships were determined using haplotype network tree in NETWORK software v5.0. Population genetic differentiation index (*F*_*ST*_) of parasites were analysed using Arlequin v3.5.

**Results:**

Sequence analysis of 11 full-length *pkmsp1* sequences along with the H-strain identified 477 (8.4%) polymorphic sites, of which 107 were singleton sites. The overall diversity observed in the full-length genes were high in comparison to its ortholog *pvmsp1* and the 4 variable domains showed extensive size variations. The nucleotide diversity was low towards the *pkmsp1*-*42* compared to the conserved domains. The 19 kDa domain was less diverse and completely conserved among isolates from Malaysian Borneo. The nucleotide diversity of isolates from Peninsular Malaysia and Thailand were higher than Malaysian Borneo. Network analysis of *pkmsp1*-*42* haplotypes showed geographical clustering of the isolates from Malaysian Borneo and grouping of isolates from Peninsular Malaysia and Thailand. Population differentiation analysis indicated high *F*_*ST*_ values between parasite populations originating from Malaysian Borneo, Peninsular Malaysia and Thailand attributing to geographical distance. Moderate genetic differentiation was observed for parasite populations from Thailand and Peninsular Malaysia. Evidence of population expansion and purifying selection were observed in all conserved domains with strongest selection within the *pkmsp1*-*42* domain.

**Conclusions:**

This study is the first to report on inter country genetic diversity and population structure of *P. knowlesi* based on *msp1*. Strong evidence of negative selection was observed in the 42 kDa domain, indicating functional constrains. Geographical clustering of *P. knowlesi* and moderate to high genetic differentiation values between populations identified in this study highlights the importance of further evaluation using larger number of clinical samples from Southeast Asian countries.

**Electronic supplementary material:**

The online version of this article (10.1186/s12936-018-2583-z) contains supplementary material, which is available to authorized users.

## Background

Malaria is a major public health threat throughout the globe and according to the World Malaria Report, 216 million cases of malaria occurred globally in 2016, with nearly a half a million deaths [1]. The simian malaria parasite *Plasmodium knowlesi* is now considered as the fifth *Plasmodium* species infecting humans and high number of cases has been reported from most Southeast Asian countries [2–6]. Highest case reports in humans due to *P. knowlesi* have been reported from Malaysia [4, 7, 8], while low number of cases have been reported from most of the Southeast Asian countries like Singapore [9], Myanmar [10], Vietnam [11], Indonesia [12, 13], Philippines [14], Cambodia [15] and Thailand [16]. Human cases of *P. knowlesi* have been on the rise since 2004 and increasing number of cases have been reported from both Peninsular Malaysia and Malaysian Borneo [4, 8, 17] and very recently from Indonesia [13, 18], thus highlighting the need for effective control measures and vaccine development. The parasite has a 24-h erythrocytic cycle and rapid increase in parasitaemia were documented to be correlated with severe malaria development in humans, which could be fatal [3, 19–21]. Though human-to-human transmission has not been reported, approximately 70–78% of malaria cases reported from Sarawak and Sabah in Malaysian Borneo are due to *P. knowlesi* [8, 19]. Recently conducted genomic and microsatellite-based investigations on *P. knowlesi* from Sarawak, Malaysian Borneo have revealed that there are 3 or more sub-clusters or sub-populations of the parasite which are associated with the two natural hosts; long-tailed (*Macaca fascicularis)* and pig-tailed (*Macaca nemestrina*) macaques [22–24]. Humans are susceptible to infections through both the associated hosts and some infections are very virulent leading to severe and fatal outcome in some patients [3, 25]. Evolutionary genes like ssrRNA and mitochondrial genes *cox 1* in *P. knowlesi* isolates from patients and macaques also showed two distinct clusters which clustered geographically to Malaysian mainland (Peninsular Malaysia) and Malaysian Borneo [26].

Extensive sequence diversity observed within candidate antigens has hindered the malaria vaccine development, thus highlighting the necessity for determining the level of polymorphisms, natural selection and population structure of the parasite populations under study. A recent genetic association study on *P. knowlesi* invasion genes *nbpxa* and *nbpxb* (normocyte binding protein xa and xb) showed that some SNPs were strongly associated with high parasitaemia and disease severity in human infections [25]. *Plasmodium knowlesi* orthologous antigens of known vaccine candidates such as Duffy binding protein (DBP), merozoite surface protein (MSP) 1, 1P and 3, normocyte binding protein xa have recently been studied from *P. knowlesi* clinical isolates [27–30]. Merozoite surface protein 1 (MSP1), a important blood stage antigen which is localized on the merozoite surface, and the C-terminus 19 kDa domain of the antigen has been found to adhere to host erythrocyte and antigenicity against the 19 kDa domain has been observed in patient serum [31–33]. In *P. knowlesi,* it is synthesized as a precursor of the 200 kDa protein during asexual stages, and through processing (proteolytic cleavage) produces four polypeptides of approximately 83, 30, 38 and 42 kDa [34]. During the invasion process, the C-terminal 42 kDa is further processed into two fragments of 33 kDa (MSP-133) and 19 kDa (MSP-119), however, only the 19 kDa fragment remains on the merozoite surface [35]. From an evolutionary point of view, all MSPs in *Plasmodium falciparum* (e.g., MSP1, MSP2, MSP4, MSP5, MSP8, and MSP10) contain an epidermal growth factor (EGF)-like domain in 1 or 2 copies at the carboxyl terminal (19 kDa domain) which is highly conserved among the family and they are attached to the membrane via glycosylphosphatidylinositol (GPI) membrane anchor [36, 37]. This conservation of the 19 kDa domain and the processing events have been observed in all human malaria species [34]. The PvMSP1-19 is found to be immunogenic and high antigenicity has been reported from patients infected with *Plasmodium vivax* [38].

Despite the fact that *pkmsp1* being an important immunogenic antigen, very few studies have genetically characterized it from the clinical isolates of Malaysia, especially from Malaysian Borneo where 80% of the natural infections in humans are reported. To date, only 12 isolates (7 from Peninsular Malaysia and 5 from Sabah, Malaysian Borneo) from Malaysia have been genetically characterized at *pkmsp*-*1*_*42*_ domain [27]. Thus, in this study firstly, 11 full-length *pkmsp*-*1* sequences from Malaysia were analysed to determine the level of diversity and natural selection at the conserved domains as demarcated by Putaporntip et al. [39]. In order to determine the intra and inter population diversity and relationship between the *msp* alleles from varied geographical isolates, *pkmsp*-*1*_*42*_ sequences from Malaysian Borneo (Sarawak and Sabah), Peninsular Malaysia and Thailand were obtained from the database (along with the H-strain). Level of sequence diversity, haplotypes circulating in each region, natural selection, phylogenetic relationships and the overall population structure were determined. Results of the present study may be beneficial for future rational design and formulation of a PkMSP1 based vaccine against *P. knowlesi,* in addition to enhancing the current knowledge pertaining to transmission dynamics of *P. knowlesi* within Malaysia and Thailand.

## Methods

### *pkmsp*-*1* and *pkmsp*-*1*_*42*_ sequence data

The *pkmsp*-*1*_*42*_ sequences were downloaded for 37 clinical isolates originating from Sarawak, 5 from Sabah, Malaysian Borneo, 11 from Peninsular Malaysia and 23 from Thailand along with the H-strain (PKNH_0728900) (Additional file 1) [24, 27, 39]. Out of these, 11 sequences (8 from Sarawak and 3 from Peninsular Malaysia) were used to characterize the full-length *pkmsp*-*1* gene (Additional file 2).

### Sequence diversity and natural selection

DnaSP v5.10 software was used to determine the sequence diversity (π), which is defined as the average number of nucleotide differences per site between two sequences [40]. Number of polymorphic sites, synonymous and non-synonymous substitutions, haplotype diversity (Hd), and haplotypes (h) within the *pkmsp1* sequences were also assessed by DnaSP software. For characterization of full-length MSP-1 sequences, only conserved domains I, III, V, VII and IX were used as the variable domains contained extensive size variations within the sequences. Graphical representation of nucleotide diversity within the 11 *pkmsp1* sequences were determined across the full-length gene with window length 100 bp and step size 25 bp using DnaSP v5.10 software. To investigate departure from neutrality, Fu and Li’s D* and F*, Tajima’s D analysis was performed [41]. Tajima’s D value is expected to be 0 when neutral.

Significantly positive Tajima’s D values imply recent population bottleneck or balancing selection, whereas negative values indicate population expansion or negative selection. The rates of synonymous (dS) and non-synonymous (dN) mutations were estimated and compared using the Z-test (*P* < 0.05) in MEGA5 incorporating the Nei and Gojobori method with the Jukes and Cantor (JC) correction and 1000 bootstrap replications [42]. Natural selection was also tested in the inter-population levels using the robust McDonald and Kreitman (MK) test with *P. vivax msp1* gene (PVX_099980) as an outgroup using DnaSP v5.10 software [43]. The test compares the ratio of the number of non-synonymous (Pn) to synonymous (Ps) polymorphic sites within a species to the numbers of non-synonymous (Dn) and synonymous (Ds) substitutions fixed sites between species per locus. Under neutrality the ratio of Dn/Ds mutations within species should be equal to Pn/Ps between species polymorphisms. However, if the ratio of fixed Dn/Ds between species is less than Pn/Ps within species, the gene is said to be under diversifying selection.

### Haplotype network

In order to determine the genealogical relationship between the haplotypes identified within the *pkmsp*-*1*_*42*_ sequences from Malaysia (Peninsular Malaysia, Sarawak and Sabah) and Thailand (obtained from human and macaques), median-joining method in NETWORK software was used.

### Population differentiation

Even though Peninsular Malaysia and Malaysian Borneo were separated by the South China Sea, samples originating from these areas were considered as one for population differentiation analysis. The ARLEQUIN software (version 3.5.1.3) [44] was used to compute pairwise differences (*F*_*ST*_) between populations, i.e., Thailand (n = 23), Malaysian Borneo (n = 42) and Peninsular Malaysia (n = 11) with 10,100 permutations. *F*_*ST*_ is a comparison of the sum of genetic variability within and between populations based on the allelic frequency differences. *F*_*ST*_ values are interpreted as no (0), low (> 0–0.05), moderate (0.05–0.15), and high (0.15–0.25) genetic differentiation.

## Results

### Genetic diversity and natural selection of full-length *pkmsp1* from Malaysia

**Fig. 1:**
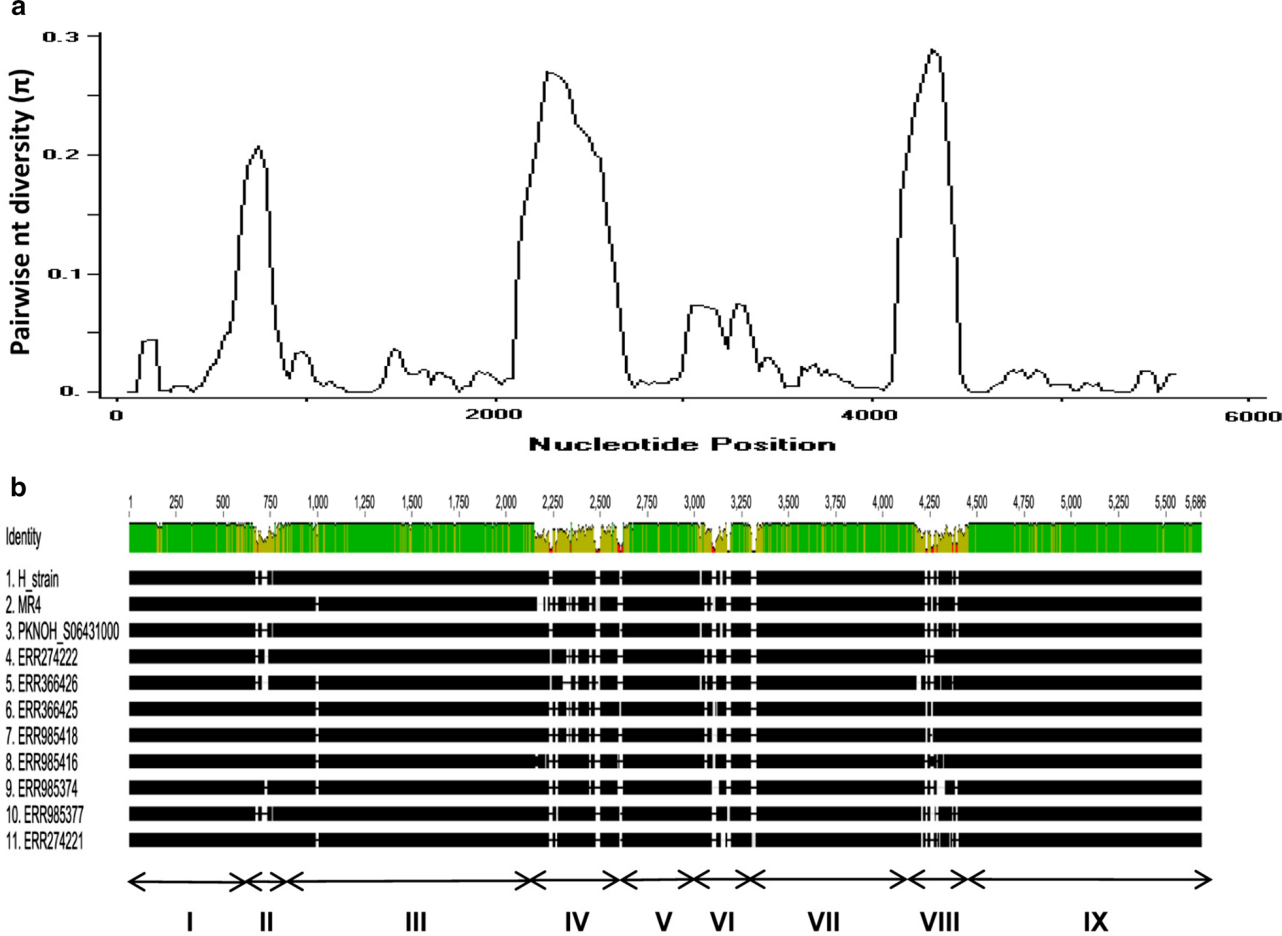
**a** Schematic diagram of *Plasmodium knowlesi* merozoite surface protein 1 (PkMSP1) domain wise nucleotide diversity within 11 Malaysian isolates. **b** Alignment of 11 *pkmsp1* isolates from Malaysia indicating the four variable domains (II, IV, VI and VIII) and conserved domains (I, III, V, VII and IX) based on Putaporntip et al. [39]. Sequence identity within the isolates are shown in dark green, SNPs as light green and alignment gaps were shown as white

The schematic structure of the *pkmsp1* gene based on the H-strain with 9 domains (5 conserved and 4 variable regions) is described in Additional file 3 with demarcation regions defined as per Putaporntip et al. [39]. Alignment and comparison of the nucleotide sequences of 11 full-length *pkmsp1* sequences revealed that there were 477 (8.4%) polymorphic sites, of which 107 were singleton sites and 370 were parsimony informative sites. Due to high number of complex repeats and insertion/deletions in the variable domains II, IV, VI and VIII, extensive size variations were observed leading to total gene length in each isolate ranging from 5403 to 5565. The overall nucleotide diversity throughout the full-length gene was π = 0.039 ± SD 0.003 (Table 1), which was higher than other merozoite invasion gene in *P. knowlesi.* The sliding window analysis of nucleotide diversity across the full-length gene is shown in Fig. 1a and a snapshot of the alignments indicating alignment gaps are shown in Fig. 1b. It was evident that high nucleotide diversity values were the result of extensive insertion/deletions and repeats motifs occurring within the *pkmsp1* variable domains II, IV, VI and VIII (Fig. 1b) of the gene. Of the 477 SNPs across the full-length gene, only 384 SNPs could be analysed (296 non-synonymous substitutions and 88 synonymous substitutions) which lead to 10 haplotypes with high haplotype diversity of 1.0 ± SD 0.04 (Table 1). Natural selection tests across the full-length gene resulted in positive value for dN–dS = 0.38 as well as Taj D and Fu and Li’s statistical test (Table 1) but not significant.

**Table 1:** Estimates of nucleotide diversity, natural selection, haplotype diversity and neutrality indices of *pkmsp1*

Domain	No. samples	SNPs	No. haplotype	Diversity ± SD		dN–dS	Codon based *z* test	Taj D	Fu and Li’s D*	Fu and Li’s F*
				Haplotype	Nucleotide					
Full-length	11	384	10	1.0 ± 0.04	0.039 ± 0.003	0.38	*P *> 0.1	0.161	0.54	0.50
I		32	9	0.978 ± 0.05	0.019 ± 0.002	− 1.14	*P *> 0.1	0.50	0.70	0.74
III		53	10	1.0 ± 0.04	0.0151 ± 0.001	− 3.88	*P *< 0.000	0.21	0.27	0.29
V		12	7	0.911 ± 0.07	0.010 ± 0.001	− 2.44	*P *< 0.01	− 0.68	− 0.72	− 0.80
VII		53	9	0.978 ± 0.05	0.019 ± 0.002	− 3.55	*P *< 0.000	− 0.23	− 0.36	− 0.37
IX (42 kDa)		27	10	1.0 ± 0.04	0.007 ± 0.0007	− 4.34	*P *< 0.000	− 0.01	− 0.05	− 0.04

*SNPs* single nucleotide polymorphisms, *SD* standard deviation

Domain wise analysis of the five conserved regions of *pkmsp1* (I, III, V, VII and IX) indicated that the nucleotide diversity towards the C-terminal (IX, 42 kDa domain) was low compared to the other conserved domains (Table 1). All the conserved domains exhibited high haplotype diversity and negative natural selection with significant statistical values for all except domain I (Table 1). The amino acid polymorphism observed within the conserved domains are listed in Additional files 4A–D.

### Inter and intra-population diversity and natural selection of *pkmsp1*-_*42*_

Alignment of 76 *pkmsp1*-_*42*_ sequences from Malaysia and Thailand along with the reference H-strain identified 74 mutations (47 synonymous and 27 non-synonymous substitutions). Of the 74 mutations, 31 were singleton sites. The nucleotide diversities of the parasite population from Peninsular Malaysia and Thailand were similar (π = 0.010 ± SD 0.001) but higher compared to Malaysian Borneo (Sarawak and Sabah) (Table 2). Extensively higher haplotype diversities were observed for all four populations due to high number of low frequency polymorphism (singletons), an indicator for parasite population expansion. The overall nucleotide diversity was found to be π = 0.009 ± SD 0.0005 and 58 were identified (Table 2). Within the 42 kDa domain, the diversity was higher towards the N terminal (33 kDa) region compared to the C-terminal (19 kDa) region (Additional file 5). Fully conserved cysteine residues towards the two EGF domains were detected in all isolates from Malaysia and Thailand, indicating conserved erythrocyte binding function. To determine the contribution of natural selection with respect to polymorphism in the *pkmsp1*-_*42*_ domain, the average difference of (dN–dS) was evaluated. The significant negative value for each of the population and together with negative values for Tajimas D and and Li and Fu’s F* and D* statistics were strongly indicative of negative or purifying selection and population expansion (Table 2). Similarly, the MK test results using *P. vivax msp1* as an outgroup also indicated that the C-terminal region (42 kDa domain) was under the influence of strong purifying selection (P < 0.01) (Table 3).

**Table 2:** Location wise nucleotide diversity, natural selection, haplotype diversity and neutrality indices of *pkmsp1*-_*42*_

Location	No. of samples	SNPs	No. haplotype	Diversity ± SD		dN–dS	Codon based *z* test	Taj D	Fu and Li’s D*	Fu and Li’s F*
				Haplotype	Nucleotide					
Malaysian Borneo (Sarawak)	37	39	32	0.989 ± 0.010	0.006 ± 0.0006	− 4.27	*P *< 0.000	− 1.07	− 1.22	− 1.33
Malaysian Borneo (Sabah)	5	20	5	1.0 ± 0.126	0.009 ± 0.002	− 3.75	*P *< 0.000	− 0.61	− 0.61	− 0.66
Peninsular Malaysia	11	28	9	0.978 ± 0.054	0.010 ± 0.001	− 3.10	*P *< 0.002	− 0.27	− 0.08	− 0.14
Thailand	23	38	14	0.937 ± 0.033	0.010 ± 0.001	− 3.21	*P *< 0.001	− 0.08	− 0.35	− 0.31
Overall	76	72	58	0.990 ± 0.005	0.009 ± 0.0005	− 4.48	*P *< 0.000	− 1.29	− 2.54**	− 2.45**

** *P *< 0.05

**Table 3:** McDonald–Kreitman tests on MSP1 of *Plasmodium knowlesi* with *P. vivax* MSP1 ortholog as outgroup species

MSP1	Polymorphic changes within *P. knowlesi*		Fixed differences between species		Neutrality index	Fisher’s exact test P-value
	Syn	NonSyn	Syn	NonSyn		
C-terminal (42 kDa)	47	27	72	84	0.49	0.01

*Syn* synonymous substitutions, *NonSyn* non synonymous substitutions

### Haplotype network analysis 76 *pkmsp1*-_*42*_

**Fig. 2:**
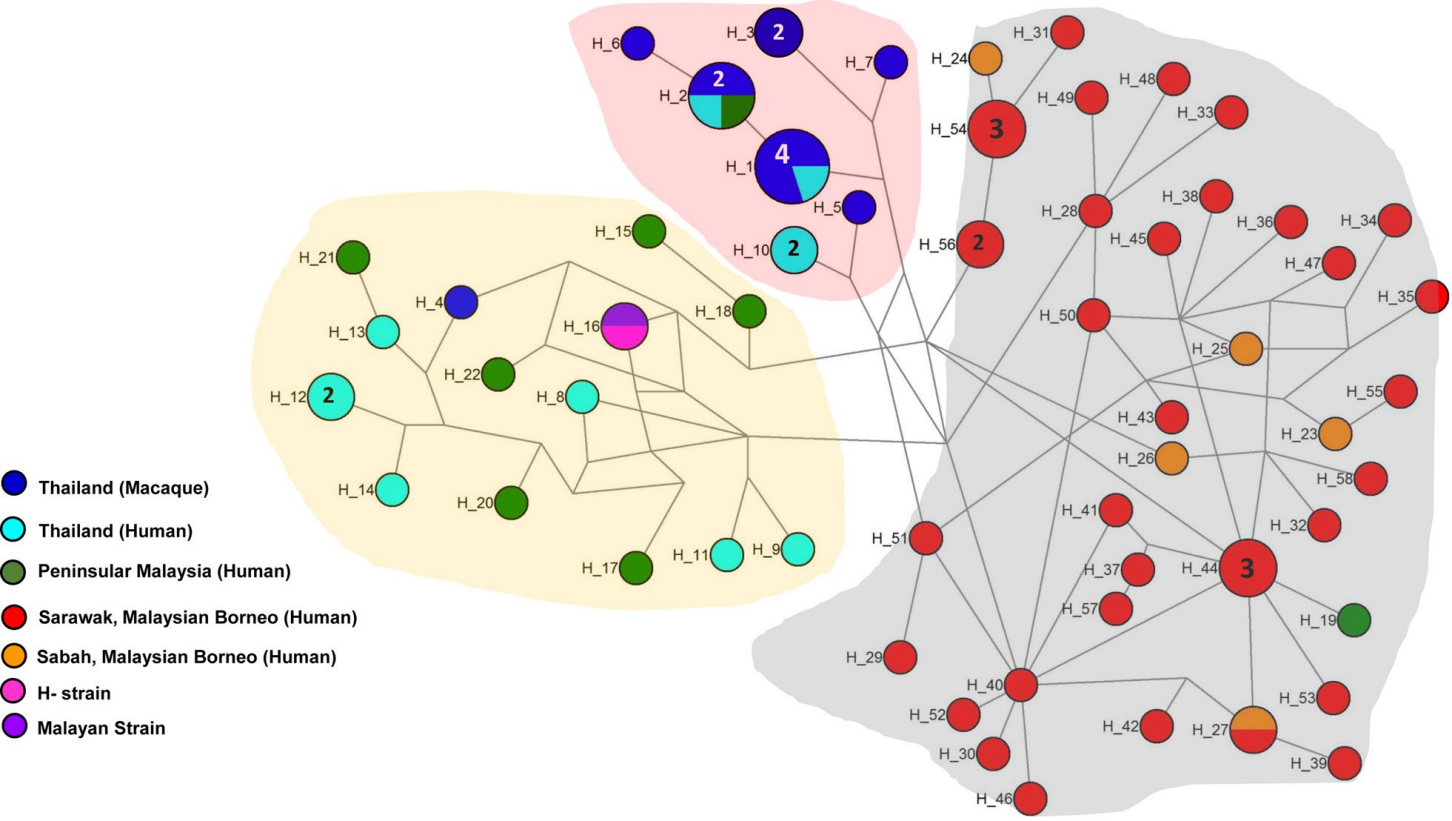
Median-joining networks of *P. knowlesi msp1*-_*42*_ haplotypes from Malaysia. The genealogical haplotype network shows the relationships among the 58 haplotypes present in the 76 sequences obtained from human and macaque samples from Thailand, Peninsular Malaysia, Sabah and Sarawak (H_n) has been used to designate a distinct haplotype number. Circle sizes represent the frequencies of the corresponding haplotype (number is indicated for those that were observed > ×1). Distances between nodes are arbitrary

Haplotype network analysis of the *pkmsp1*-_*42*_ using median-joining method showed that all haplotypes from Sarawak (Malaysian Borneo) and Sabah (Malaysian Borneo) grouped together indicating geographical clustering of parasites originating from Malaysian Borneo (Fig. 2). Most macaque isolates from Thailand formed a unique group along with shared haplotypes of human and macaques (H_1, H_2) from Thailand (Fig. 2). H_2 was shared between human and macaque from Thailand and Peninsular Malaysia indicating common origin of the parasites. Most haplotypes from Peninsular Malaysia grouped with haplotypes from Thailand (human) indicating common ancestry of the parasites (Fig. 2). However, one haplotype from Peninsular Malaysia (H_19) also grouped along of the isolates from Malaysian Borneo (Fig. 2). The reference H-strain and the Malayan Strain also grouped along with isolates from Peninsular Malaysia (Fig. 2). It is interesting to note that the two distinct sub-populations of *P. knowlesi* reported in clinical samples from Sarawak in other MSP antigens [30] were not observed in the phylogenetic network analysis of the haplotypes in this study.

### Amino acid haplotypes of 76 PkMSP142

**Fig. 3:**
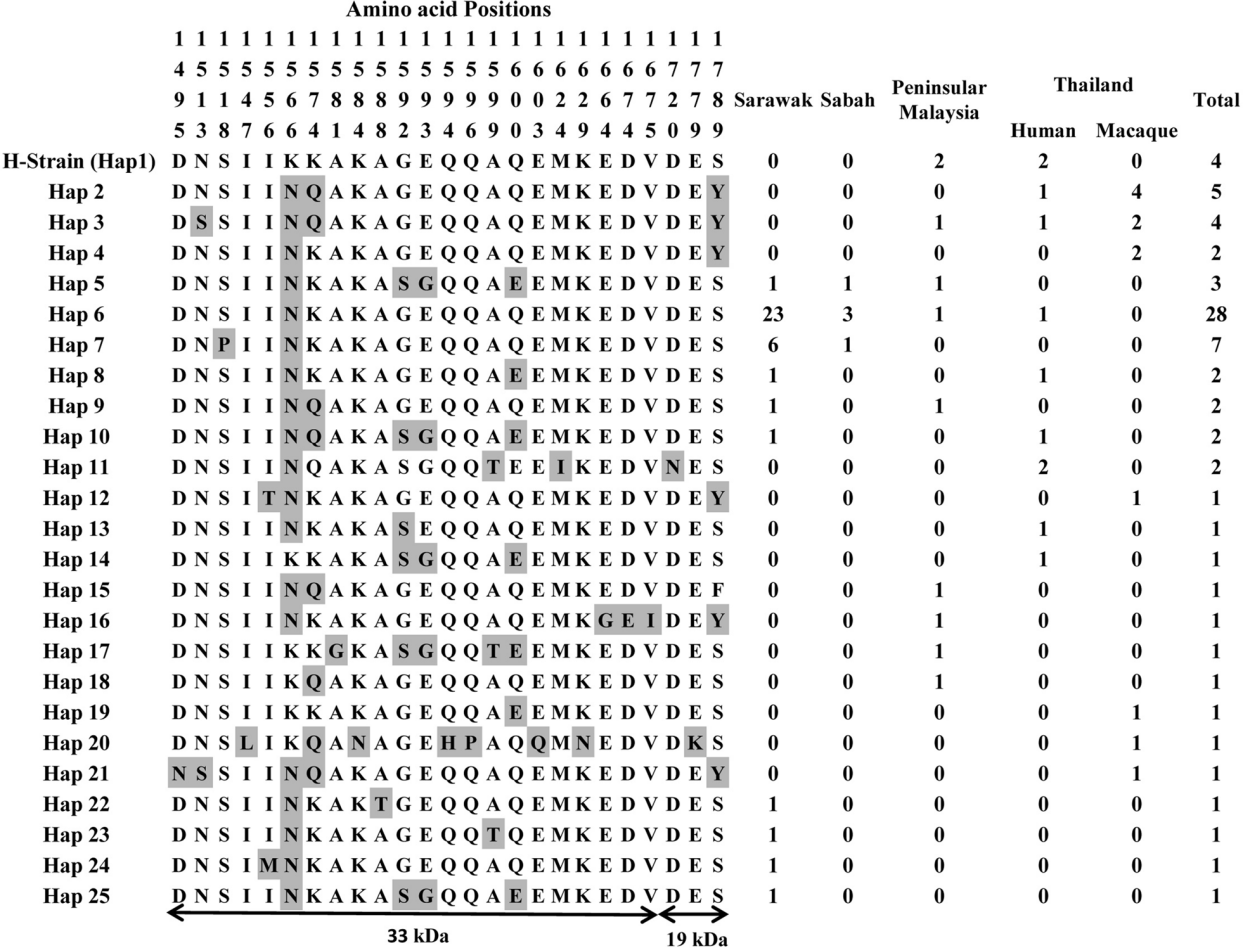
PkMSP1-42 haplotypes observed among Malaysian and Thailand isolates. The polymorphic amino acid sites falling within the 33 kDa and the 19 kDa domain are denoted through the arrow heads below and the residues are shaded in grey within each haplotypes. Numbers above the haplotypes indicate amino acid positions with respect to the reference H-strain. Frequencies of each haplotypes identified from Malaysia (Peninsular Malaysia, Sarawak and Sabah) and Thailand are represented in numerical. The number of sample from each site/host are shown along with the haplotypes

Alignment of 76 PkMSP1-42 amino acid sequences identified 25 haplotypes (Fig. 3). Majority of the share haplotypes were observed within Haplotype 6 (Hap 6) which had isolates from Sarawak (n = 23), Malaysian Borneo, Sabah (n = 3) and one each from Peninsular Malaysia and Thailand. Within the haplotypes, the amino acid polymorphism was higher towards the 33 kDa domain compared to the 19 kDa domain (Fig. 3). Variations in the 19 kDa domain were observed only at 3 amino acid positions (D127N, E177K and S178Y), of which, mirror allele frequency of > 10% was observed only S178Y site. Shared haplotypes between Thailand, Peninsular Malaysia were observed in Haplotype 1 and 3 however, with very low frequency (Fig. 3). The isolates from Malaysian Borneo had completely conserved 19 kDa domain and the domain resembled the H-strain sequence.

### Population differentiation *pkmsp1*-_*42*_

Pairwise population differentiation index *F*_*ST*_ values using ARLEQUIN software demonstrated high genetic differentiation between the parasite populations originating from Peninsular Malaysia and Malaysian Borneo (*F*_*ST*_ = 0.237, *P* < 0.000) (Table 4), which most likely attributed to geographical distance between the two regions due to the South China Sea separating them. Similarly high *F*_*ST*_ values were observed for parasites originating from Thailand and Malaysian Borneo (Table 4) however, moderate level of genetic differentiation was observed between parasites of Peninsular Malaysia and Thailand (*F*_*ST*_ = 0.071, *P* < 0.05) (Table 4). These results indicate that the transmission of the parasites may be confined to each region.

**Table 4:** Population differentiation values (*F*_*ST*_) for *pkmsp1*-_*42*_ from Malaysia and Thailand

Location	*F*_*ST*_ values*		
	Malaysian Borneo	Peninsular Malaysia	Thailand
Malaysian Borneo	–	–	–
Peninsular Malaysia	0.237**	–	–
Thailand	0.233**	0.071*	–

** P < 0.0000, * P < 0.05

## Discussion

The PkMSP1-42 has been studied as a novel vaccine candidate and generation of protective immune response from patient serum using recombinant expressed proteins has been reported [45]. However, very limited clinical isolates have been characterized genetically at this domain to evaluate the polymorphisms at the population level, which is most critical in terms of feasibility of a vaccine candidate. Thus, purpose of the current study was to genetically characterize the *pkmsp1* gene from Malaysia and assess the level of genetic diversity, natural selection acting upon the full-length PkMSP1 and 42 kDa domain. Sequence alignment of 11 full-length sequences of *pkmsp1* genes from Malaysia illustrated that it has extensive polymorphisms across the gene, mostly due to the variable regions II, IV, VI and VIII. Among the conserved domains, the C-terminal domain IX (42 kDa) had the lowest nucleotide diversity, a phenomenon observed in all MSPs specifically in the 19 kDa domain [27, 30, 39]. Interestingly, all of the conserved domains I, III, V, VII and IX exhibited high haplotype diversity and it is due to the presence of high number of singleton sites low frequency polymorphisms (Si = 107). Presence of high number of low frequency polymorphism was observed in a number of merozoite invasions genes in *P. knowlesi* from clinical isolates [22, 25, 29]. The presence of 107 singleton variable sites detected across the full-length gene revealed that new and rare variants were present, suggesting population expansion but only domains V, VII and IX with negative values for Li and Fu’s D* and F*. However, overall, the full-length gene did not show significant values for Li and Fu’s statistic probably due the presence of hyper variable domains. The negative selection pressure and population expansion observed in each of the conserved domains indicate that the parasite population might be under strong functional constrains.

Inter population diversity indices based on the *Pkmsp1*-_*42*_ indicated that irrespective of geographical origin of the parasite populations, the haplotype diversities were of similar range, implying no population wise variations despite the high number of cases in Malaysian Borneo. Moderately higher nucleotide diversity was observed for samples originating from Peninsular Malaysia and Thailand. It is interesting to note that despite the presence of extensive polymorphism and high nucleotide diversity in other domains of the gene, the 42 kDa domain had low diversity in the intra-population level (π = 0.009). Similar low levels of intra-population diversities have been observed for isolates from Thailand [39] and other apical proteins in *P. knowlesi* [46]. Significant negative/purifying selection was observed within the 42 kDa domain, denoting functional constraints were present within the parasite populations of all the four geographical locations in this study. All statistics like Taj D, Li and Fu’s D* and F* values were negative indicating population expansion and negative natural selection within the 42 kDa domain. Within 76 PkMSP1-42 sequences, only 25 amino acid haplotypes were identified of which highest cluster was from Sarawak, Malaysian Borneo (Hap 6, n = 23) indicating low variations within isolates from Sarawak compared to other regions. Comparison of amino acid and nucleotide haplotypes from each region indicated that almost each population had similar number of samples, i.e. Peninsular Malaysia sample size (n = 11, 9 nucleotide haplotypes vs 9 amino acid haplotypes); for Thailand sample size (n = 23, 14 nucleotide haplotypes vs 13 amino acid haplotypes), and Sabah (n = 5, 5 nucleotide haplotypes vs 3 amino acid haplotypes). However, for Sarawak, there were 32 nucleotide haplotypes vs 10 amino acid haplotypes with a sample size n = 37. This was probably due to higher number of singleton sites in samples from Sarawak indicating population expansion (higher negative values for Li and Fu’s F* and D*). It is interesting to note that the polymorphisms towards the 19 kDa domain was limited to only one site (S178Y) with minor allele frequency > 10%. Also, variations within the 19 kDa domain were mostly observed within isolates originating from Peninsular Malaysia and Thailand. All isolates originating from Malaysian Borneo had conserved 19 kDa domains indicating conserved functional activity.

The median-joining based haplotype network analysis did not show separation of the *P. knowlesi msp1*-_*42*_ into two sub-populations as observed for other invasion genes such as *nbpxa, msp1p, dbpII* etc. where deep dimorphism was noted due to host associated factors [22, 25, 30, 47, 48]. Instead, the MSP1 haplotypes revealed geographical clustering, indicating an evolutionary conservation based on sample origin. Similar feature was observed in other evolutionary genes, including but not limited to *PkssrRNA* and *Pkmt* [26]. However, one haplotype from Peninsular Malaysia grouped together with haplotypes from Malaysian Borneo, signifying historical common origin which may be attributed to evolution of the parasites and apparent sea level rise during ice age leading to separation [26]. However, higher number of samples from Peninsular Malaysia and Thailand would be necessary for accurate assessment.

Population differentiation analyses also showed high genetic differentiation between parasite populations originating from Peninsular Malaysia and Malaysian Borneo, which can be attributed to geographical separation of the populations due to the South China Sea. Similarly, high *F*_*ST*_ values were also observed for parasite populations from Thailand and Malaysian Borneo. However, moderate genetic differentiation was observed for parasite populations from Thailand and Peninsular Malaysia probably because of shared landmass. These observations may suggest human susceptibility to infection with any one of the *P. knowlesi* populations circulating in these regions. It is also not known if some are more susceptible than others. However, higher number of human and macaque samples from Peninsular Malaysia as well as Thailand would be necessary to accurately ascertain the transmission routes of *P. knowlesi*.

## Conclusion

The present study investigates genetic diversity, natural selection and population structure of the *pkmsp1* gene from three different regions with different *P. knowlesi* transmission rates. High number of haplotypes and haplotype diversity was identified in each regions and the C-terminal 42 kDa region appeared to be under strong purifying selection and undergoing population expansion. Phylogenetic network analysis indicated geographical clustering of the parasites specifically from Malaysian Borneo and grouping of parasites from Peninsular Malaysia and Thailand. Future studies should investigate the diversity of PkMSP1 among *P. knowlesi* isolates from all Southeast Asian countries.

## Additional files

**Additional file 1.** Geographical origin of samples used in this study.

**Additional file 2.** Accession number of PkMSP1 sequences used in the study and their geographical origin.

**Additional file 3.** Schematic diagram of *Plasmodium knowlesi* MSP1 protein domains. Each box in the schematic diagram is representative of the various conserved and variable domains. Domain coordinates have been marked following Putaporntip et al. [39]. Conserved domains I, III, V, VII, and IX are in shaded background whereas variable domains II, IV, VI, and VIII are in dotted background. Signal peptide, trans-membrane domain and Epidermal Growth Factor have been abbreviated as SP, TM and EGF, each respectively.

**Additional file 4.** Amino acid alignment of PkMSP1 (A) Domain I, (B) Domain III, (C) Domain V and (D) Domain VII between Thailand (n = 23) and Malaysian (n = 11) isolates. Period and hyphen represents identical amino acids and deletions, respectively. Thailand; AEQ01041-AEQ01055 and AFR68690–AFR68697. Malaysia; deduced amino acids ERR274221, ERR274222, ERR366425, ERR366426, ERR985374, ERR985377, ERR985416, ERR985418, and P_Malaysia_2 along with H-strain (CAQ39354). Others [M] and Others [T] refers to observed sequential variations within Malaysian or Thailand isolates, respectively.

**Additional file 5.** Graphical representation of nucleotide diversity of PkMSP1 at the 33 kDa and 19 kDa domains.

## Abbreviations

MSP1: merozoite surface protein 1
kDa: kilodalton
