# Supplementary figures and images for: Within-population genetic diversity and population structure of Plasmodium knowlesi merozoite surface protein 1 gene from geographically distinct regions of Malaysia and Thailand
Source: Malar J. 2018 Nov 29;17:442. doi: 10.1186/s12936-018-2583-z (PMC6267868; doi:10.1186/s12936-018-2583-z)

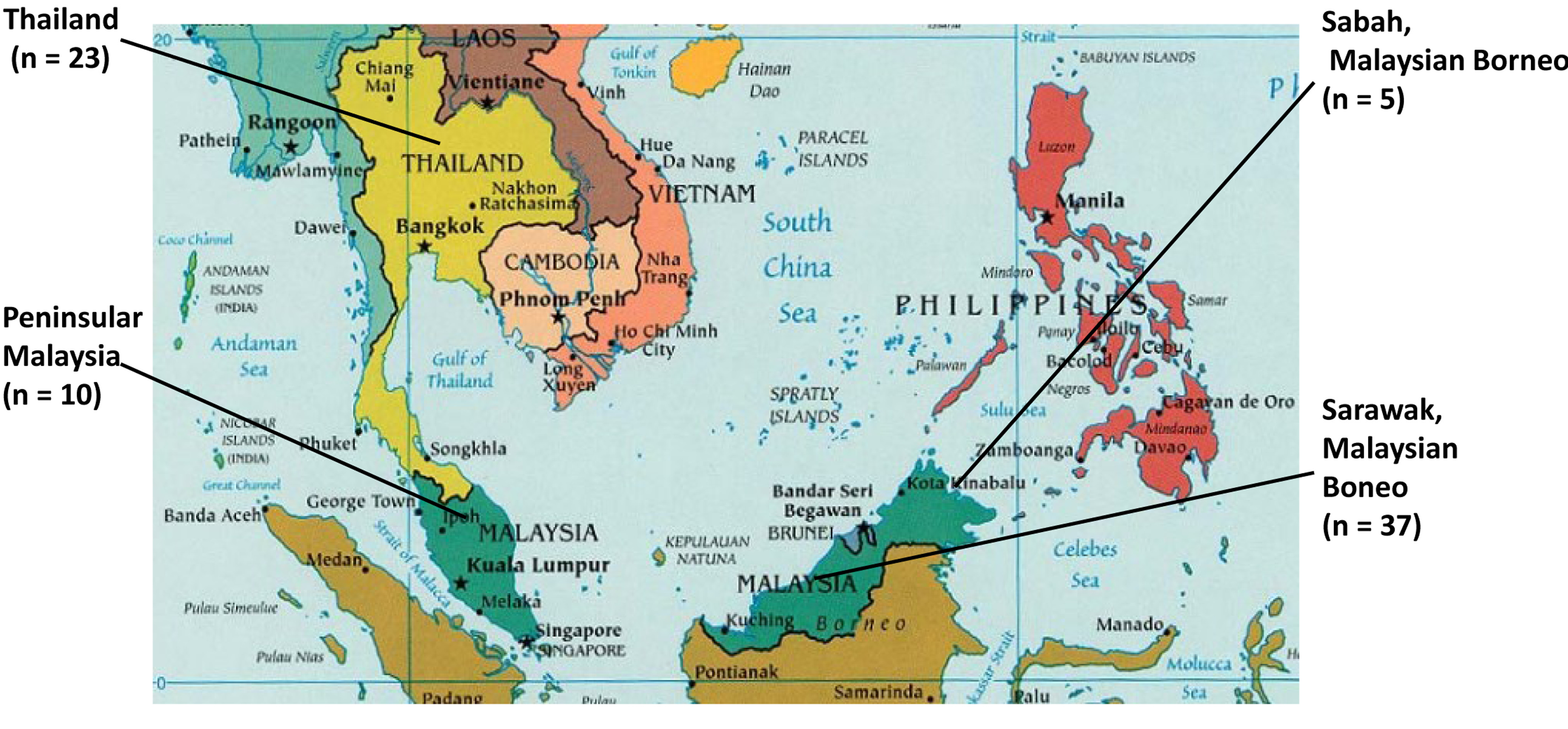

Supplement: Supplementary file 1 — Additional file 1. Geographical origin of samples used in this study. [file 12936_2018_2583_MOESM1_ESM.jpg]

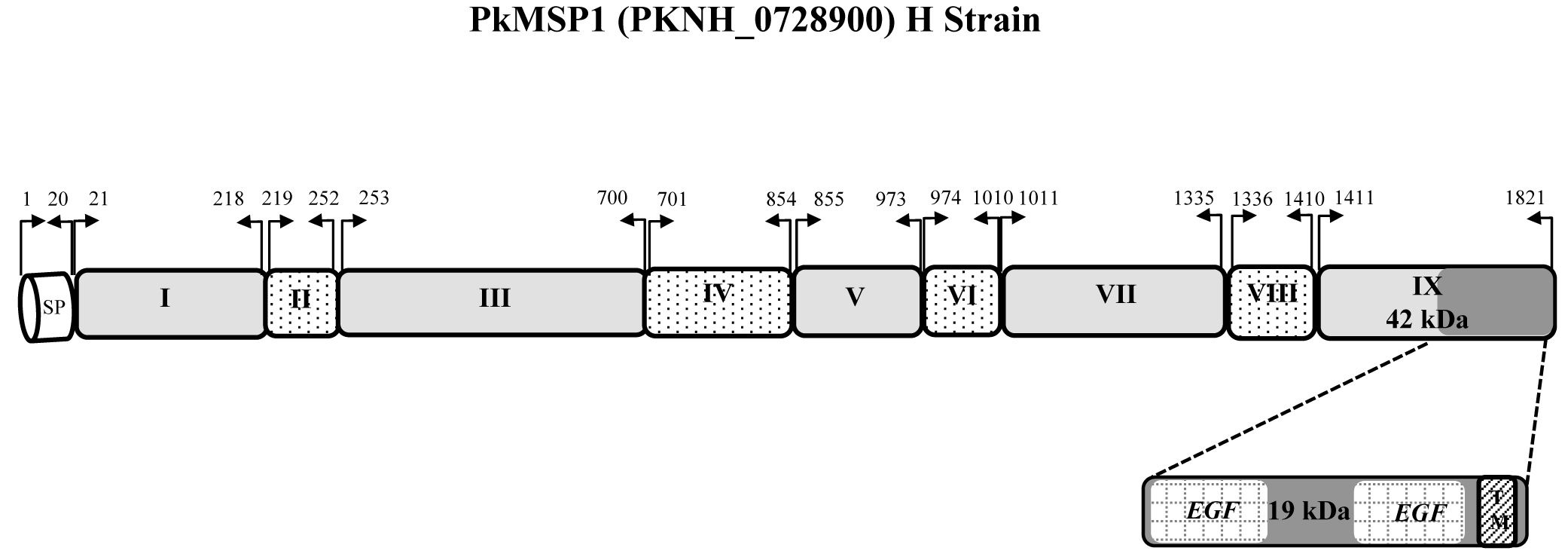

Supplement: Supplementary file 3 — Additional file 3. Schematic diagram of Plasmodium knowlesi MSP1 protein domains. Each box in the schematic diagram is representative of the various conserved and variable domains. Domain coordinates have been marked following Putaporntip et al. [39]. Conserved domains I, III, V, VII, and IX are in shaded background whereas variable domains II, IV, VI, and VIII are in dotted background. Signal peptide, trans-membrane domain and Epidermal Growth Factor have been abbreviated as SP, TM and EGF, each respectively. [file 12936_2018_2583_MOESM3_ESM.jpg]

A


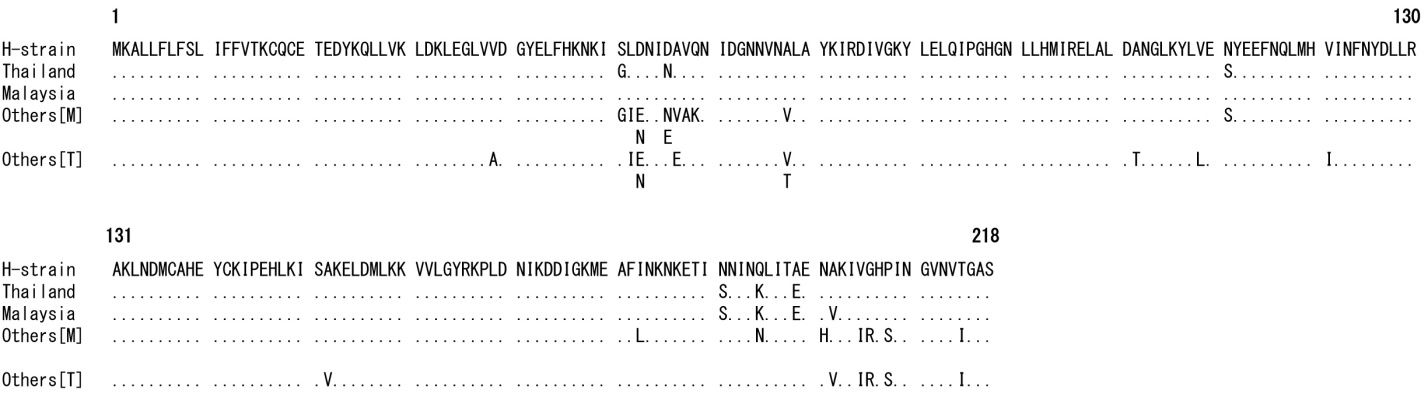


B


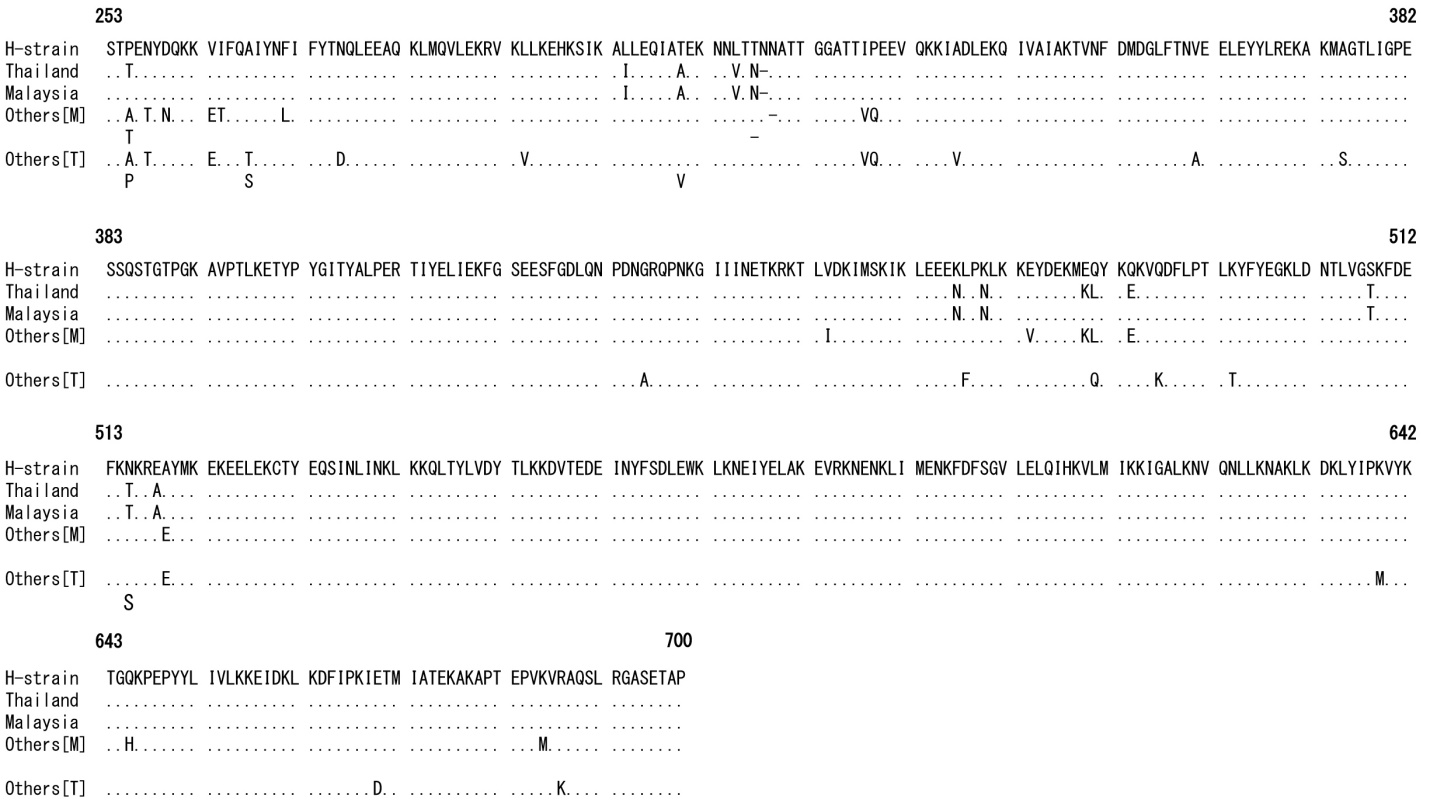


C


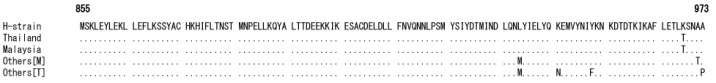


D


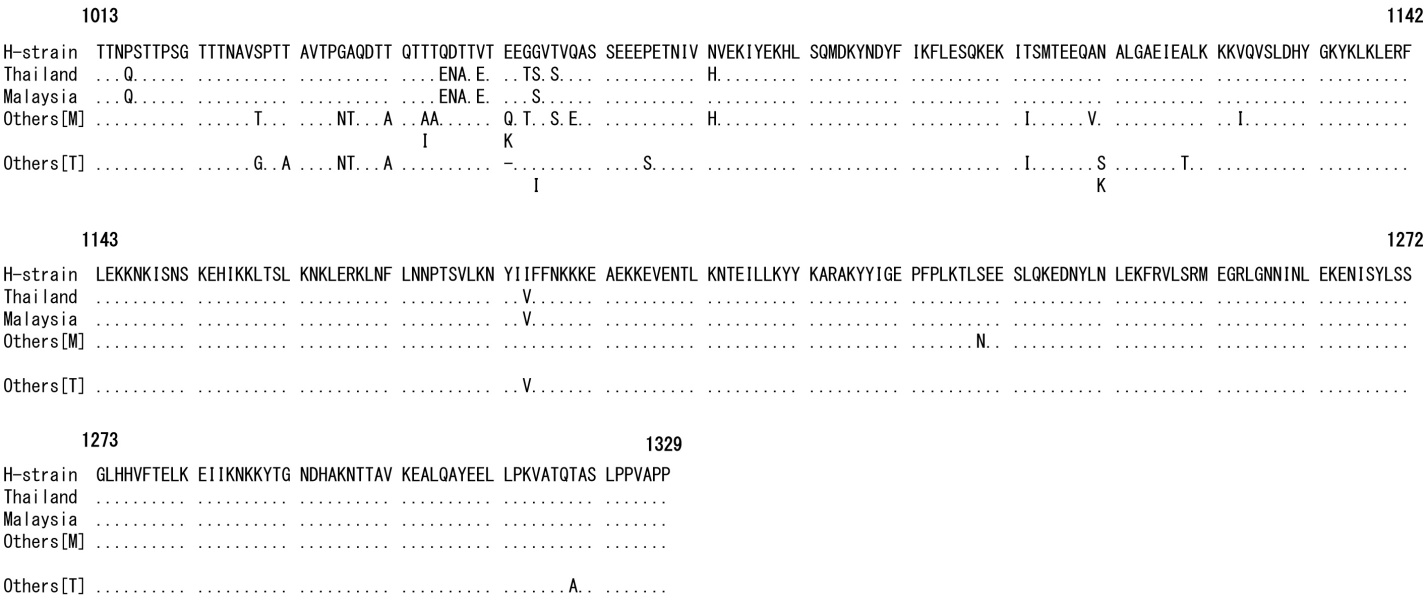

Supplement: Supplementary file 4 — Additional file 4. Amino acid alignment of PkMSP1 (A) Domain I, (B) Domain III, (C) Domain V and (D) Domain VII between Thailand (n = 23) and Malaysian (n = 11) isolates. Period and hyphen represents identical amino acids and deletions, respectively. Thailand; AEQ01041-AEQ01055 and AFR68690–AFR68697. Malaysia; deduced amino acids ERR274221, ERR274222, ERR366425, ERR366426, ERR985374, ERR985377, ERR985416, ERR985418, and P_Malaysia_2 along with H-strain (CAQ39354). Others [M] and Others [T] refers to observed sequential variations within Malaysian or Thailand isolates, respectively. [file 12936_2018_2583_MOESM4_ESM.docx]

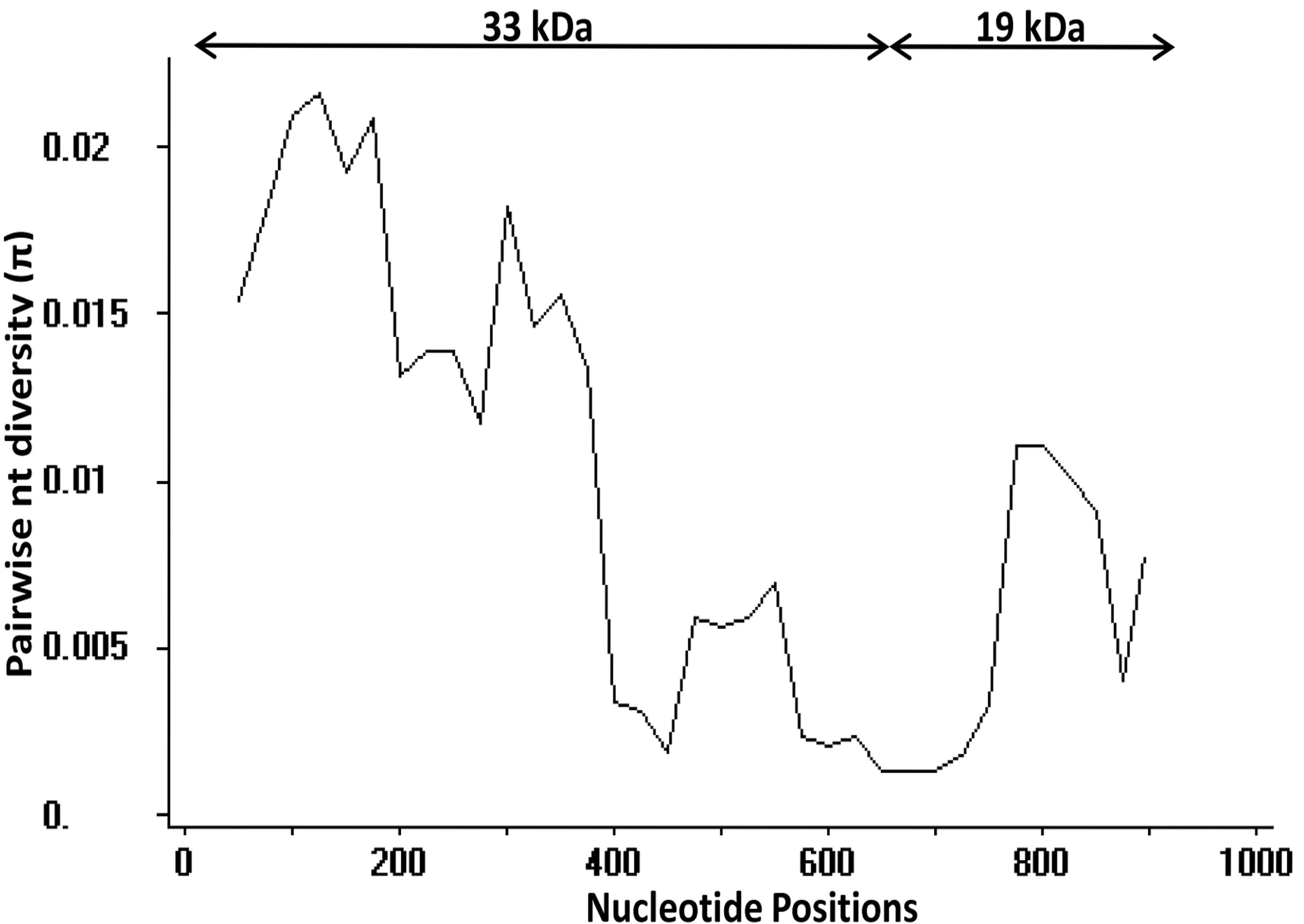

Supplement: Supplementary file 5 — Additional file 5. Graphical representation of nucleotide diversity of PkMSP1 at the 33 kDa and 19 kDa domains. [file 12936_2018_2583_MOESM5_ESM.jpg]
