# Supplementary material for: Within-population genetic diversity and population structure of Plasmodium knowlesi merozoite surface protein 1 gene from geographically distinct regions of Malaysia and Thailand
Source: Malar J. 2018 Nov 29;17:442. doi: 10.1186/s12936-018-2583-z (PMC6267868; doi:10.1186/s12936-018-2583-z)
Supplement: Supplementary file 2 — Additional file 2. Accession number of PkMSP1 sequences used in the study and their geographical origin. [file 12936_2018_2583_MOESM2_ESM.docx]

**Additional file 2 Table S1. Study samples and origin**

| **No.** | **Sample ID** | **Area** |
| --- | --- | --- |
| 1 | **ERR274221** | Malaysia(B) |
| 2 | **ERR274222** | Malaysia(B) |
| 3 | **ERR366425** | Malaysia(B) |
| 4 | **ERR366426** | Malaysia(B) |
| 5 | **ERR985374** | Malaysia(B) |
| 6 | ERR985376 | Malaysia(B) |
| 7 | **ERR985377** | Malaysia(B) |
| 8 | ERR985378 | Malaysia(B) |
| 9 | ERR985379 | Malaysia(B) |
| 10 | ERR985380 | Malaysia(B) |
| 11 | ERR985381 | Malaysia(B) |
| 12 | ERR985382 | Malaysia(B) |
| 13 | ERR985383 | Malaysia(B) |
| 14 | ERR985384 | Malaysia(B) |
| 15 | ERR985385 | Malaysia(B) |
| 16 | ERR985386 | Malaysia(B) |
| 17 | ERR985387 | Malaysia(B) |
| 18 | ERR985388 | Malaysia(B) |
| 19 | ERR985390 | Malaysia(B) |
| 20 | ERR985392 | Malaysia(B) |
| 21 | ERR985393 | Malaysia(B) |
| 22 | ERR985394 | Malaysia(B) |
| 23 | ERR985395 | Malaysia(B) |
| 24 | ERR985396 | Malaysia(B) |
| 25 | ERR985397 | Malaysia(B) |
| 26 | ERR985404 | Malaysia(B) |
| 27 | ERR985405 | Malaysia(B) |
| 28 | ERR985406 | Malaysia(B) |
| 29 | ERR985407 | Malaysia(B) |
| 30 | ERR985408 | Malaysia(B) |
| 31 | ERR985409 | Malaysia(B) |
| 32 | ERR985410 | Malaysia(B) |
| 33 | ERR985411 | Malaysia(B) |
| 34 | **ERR985416** | Malaysia(B) |
| 35 | ERR985417 | Malaysia(B) |
| 36 | **ERR985418** | Malaysia(B) |
| 37 | SRR2222335 | Malaysia(P) |
| 38 | SRR3135172 | Malaysia(P) |
| 39 | **Malayan Strain Pk1A PKNOH_S06431000** | Malaysia(P) |
| 40 | **H-strain(PKNH_0728900)** | Malaysia(P) |
| 41  42 | JF837339  JF837340 | Thailand(m)  Thailand(m) |
| 43  44  45  46  47  48  49  50  51  52  53  54  55  56  57  58  59  60  61  62  63  64  65  66  67  68  69  70  71  72  73  74  75 | JX046794  JF837341  JX046797  JF837344  JX046793  JX046795  JX046798  JX046796  JX046792  JX046791  JF837342 JF837343  JF837345  JF837346  JF837347  JF837348  JF837349  JF837350  JF837351  JF837352  JF837353  KX881364  KX881363  KX881365  KX881366  KX881370  KX881371  KX881367  KX894506  KX881368  KX881369  KX894507  KX894505 | Thailand(m)  Thailand(m)  Thailand(m)  Thailand(m)  Thailand(m)  Thailand(m)  Thailand(m)  Thailand(m)  Thailand(m)  Thailand(m)  Thailand(m)  Thailand  Thailand  Thailand  Thailand  Thailand  Thailand  Thailand  Thailand  Thailand  Thailand  Malaysia(P)  Malaysia(P)  Malaysia(P)  Malaysia(P)  Malaysia(P)  Malaysia(P)  Malaysia(P)  Malaysia(BS)  Malaysia(BS)  Malaysia(BS)  Malaysia(BS)  Malaysia(BS) |
| 76 | **MR4H** |  |

(P): Peninsular; (B): Sarawak, Malaysian Borneo; (m): macaque; (BS): Sabah, Malaysian Borneo

Isolates in bold were used for full length characterisation of *pkmsp1* gene.
